# Maternal Vitamin D Status and Adverse Birth Outcomes in Children from Rural Western Kenya

**DOI:** 10.3390/nu8120794

**Published:** 2016-12-07

**Authors:** Eunice N. Toko, Odada P. Sumba, Ibrahim I. Daud, Sidney Ogolla, Maxwel Majiwa, Jesse T. Krisher, Collins Ouma, Arlene E. Dent, Rosemary Rochford, Saurabh Mehta

**Affiliations:** 1 School of Biological and Physical Sciences, Maseno University, Kisumu, Kenya; eunicetoko@gmail.com (E.N.T.); sidneyogolla@yahoo.com (S.O.); collinouma@yahoo.com (C.O.); 2 Center for Global Health Research, Kenya Medical Research Institute, Kisumu, Kenya; odadakasumba@gmail.com (O.P.S.); ibrayed@yahoo.com (I.I.D.); mmajiwa@uw.edu (M.M.); 3 Jomo Kenyatta University of Agriculture and Technology, Nairobi, Kenya; 4 Division of Nutritional Sciences, Cornell University, 314 Savage Hall, Ithaca, NY 14853, USA; jtk93@cornell.edu; 5 African Institute for Development Policy, Nairobi, Kenya; 6 Case Western Reserve University, Cleveland, OH 44106, USA; aed9@case.edu; 7 SUNY Upstate Medical University, Syracuse, NY 13210, USA; 8 Department of Immunology and Microbiology, University of Colorado, Denver, Aurora, CO 80045, USA

**Keywords:** maternal vitamin D status, pregnancy outcomes, stunting, Africa, malaria and helminth infections

## Abstract

Maternal plasma 25-hydroxyvitamin D (25(OH)D) status and its association with pregnancy outcomes in malaria holoendemic regions of sub-Saharan Africa is poorly defined. We examined this association and any potential interaction with malaria and helminth infections in an ongoing pregnancy cohort study in Kenya. The association of maternal plasma 25(OH)D status with pregnancy outcomes and infant anthropometric measurements at birth was determined in a subset of women (*n* = 63). Binomial and linear regression analyses were used to examine associations between maternal plasma 25(OH)D and adverse pregnancy outcomes. Fifty-one percent of the women had insufficient (<75 nmol/L) and 21% had deficient (<50 nmol/L) plasma 25(OH)D concentration at enrollment. At birth, 74.4% of the infants had insufficient and 30% had deficient plasma 25(OH)D concentrations, measured in cord blood. Multivariate analysis controlling for maternal age and body mass index (BMI) at enrollment and gestational age at delivery found that deficient plasma 25(OH)D levels were associated with a four-fold higher risk of stunting in neonates (*p* = 0.04). These findings add to the existing literature about vitamin D and its association with linear growth in resource-limited settings, though randomized clinical trials are needed to establish causation.

## 1. Introduction

The importance of vitamin D and its regulation of calcium and phosphorus in ensuring optimal pregnancy outcomes has been examined ever since its discovery in the early 1900s [1,2,3]. Many of these studies initially focused on the prevention of osteomalacia and eclampsia [4,5,6,7]. For instance, earlier studies have shown that a diet supplemented with calcium, phosphorus, and vitamin D could treat osteomalacia in China and India [2].

Adequate provision of vitamin D during pregnancy is essential for bone mineralization of the developing fetus [5,8]. It is estimated that approximately 25–30 g of calcium are transferred to the fetal skeleton by the end of pregnancy, most of it during the last trimester [9,10,11]. The role of vitamin D in this process may be indicated by an increase in the concentrations of the active form, 1.25-dihydroxyvitamin D—50%–100% over the non-pregnant state in the second trimester and over 100% in the third trimester [12,13]. The increase in concentrations potentially accounts for changes such as greater transfer of calcium to the fetus [5,6]. Poor vitamin D status may lead to severe unwanted pregnancy outcomes involving either skeletal (growth restriction) or non-skeletal health complications, including pre-eclampsia, gestational diabetes, preterm birth, and low birth weight [13,14,15,16]. 

Typically, in vivo vitamin D micronutrient metabolism is mainly dependent on direct exposure of skin to ultraviolet (UV) rays, which is required for the isomerization of endogenous 7-dehydrocholesterol in the formation of cholecalciferol [1,3,4]. However, despite adequate and stable sunny climatic conditions in equatorial countries, studies have reported insufficient circulating 25-hydroxyvitamin D (25(OH)D) concentrations (<75 nmol/L) in infants [17,18], pregnant and lactating mothers, and generally in adults [19]. Variations in 25(OH)D concentrations in these populations have been attributed to varying levels of sun exposure, predominantly due to changing life styles. This may, in part, come into play following increased urbanization, seasonal climatic changes, skin pigmentation, clothing style, use of sunscreen, and dietary habits [20]. Additionally, vitamin D deficiency has been shown to play a role in susceptibility to bacterial and viral infections, such as tuberculosis [21] and human immunodeficiency virus (HIV) [22]. Maternal vitamin D deficiency and parasitic infections during pregnancy have also been associated with an increased risk of anemia and iron deficiency [23].

In this study, we measured maternal plasma 25(OH)D through the different trimesters and in the infant’s cord blood at delivery in a cohort from western Kenya, where malaria transmission is high. The infants’ anthropometric measurements were taken at birth. In addition, parasitic infection status and pregnancy outcomes were determined, and their association with maternal plasma 25(OH)D was analyzed.

## 2. Materials and Methods

### 2.1. Study Design and Setting

This longitudinal study was conducted at Chulaimbo Sub-district hospital in Western Kenya from June 2011 to July 2012. Chulaimbo Sub-district hospital is a rural public health facility located in Kisumu County, Kenya, within the Lake Victoria basin—an area distinctly characterized by holoendemic malaria transmission [24]. The facility serves approximately 19,230 patients annually. The Chulaimbo Sub-district hospital catchment zone is densely populated, and therefore, many residents own small parcels of land that allow them to practice subsistence farming with fishing as the main economic activity.

This study was approved by both Kenya Medical Research Institute and State University of New York (SUNY) Upstate Medical University Ethical Review Boards. Written informed consent was obtained from all the mothers before participation.

### 2.2. Study Participants

As previously described [25], pregnant women of all gravidities, less than twenty six weeks gestation, who were HIV-1 uninfected, residing within a radius of 10 km from the hospital, and were willing to participate in the study were recruited from June to November 2011. Gestational age was assessed by measurement of fundal height and history of the last menstrual period. The women were evaluated in active monthly follow-up through antenatal clinic (ANC) visits until delivery. A total of 99 mothers delivered at the hospital.

### 2.3. Anthropometric Measurements

Maternal anthropometric measurements (height and weight) were determined at enrollment, and infants’ weight, length, and head circumference were recorded at birth using Seca mechanical measuring scales (Seca GMBH & Co., Hamburg, Germany; model number: 762).

### 2.4. Blood Sample Collection, Processing, and Preservation

At enrollment and subsequent ANC visits, thin and thick blood smears were prepared for malaria diagnosis by microscopy; approximately 200 μL of maternal venous blood was collected by venipuncture into ethylenediaminetetraacetic acid (EDTA) microtainer tubes (BD, Franklin Lakes, NJ, USA) for malaria diagnosis by quantitative polymerase chain reaction (qPCR) as described [25]. Hemoglobin (Hb) was measureed using a portable β-hemoglobin photometer (Hemocue AB, Angelholm, Sweden), and circulating 25(OH)D concentrations were assessed using enzyme immunoassay (IDS EIA kit, Immunodiagnostic Systems, Boldon, UK). The neonates’ cord blood was also collected from the umbilical vein aseptically into EDTA microtainer tubes (BD, Franklin Lakes, NJ, USA) as described earlier [26] and used for vitamin D EIA assays. All samples were transported to the Kenya Medical Research Institute (KEMRI) laboratory located at the Center for Global Health Research in Kisumu for processing within approximately one hour of blood collection. The plasma fraction was separated from whole blood by centrifugation (Thermo Fisher Scientific, Jouan C412, Waltham, MA, USA) at 1500 r.p.m., and aliquots were stored at −80 °C until use for vitamin D quantification by ELISA. To ensure analytical reliability, KEMRI has been participating in the Vitamin D External Quality Assurance Scheme (DEQAS) program [27], and a sampling from the January 2016 report shows that the results from the IDS-EIA has high correlation with the standard used. The intra-assay coefficient of variability (CV) averaged 2.39% ± 2.36% across seven plates analyzed.

### 2.5. Standard of Care

All mothers were tested for HIV-1 infection status as part of Mother-to-Child-Transmission (MTCT) of HIV programs in accordance with Kenya Ministry of Health (MOH) national guidelines. This being a malaria holoendemic area, malaria status was confirmed by microscopy and quantitative real-time polymerase chain reaction (qRT-PCR). Those diagnosed with the infection were treated with artemether (20 mg) and lumefantrine (120 mg), a first line anti-malarial drug recommended for non-severe malaria by the Kenyan MOH. During the second trimester of the gestation period, all the expectant mothers were given sulfadoxine/pyrimethamine (SP) for malaria prophylaxis, as recommended by the Kenyan MOH. At the first ANC visit, stool samples were collected and analyzed for the presence of helminthes; infected mothers were treated with albendazole in accordance with the Kenyan MOH. Vitamin D supplementation is not part of the Kenyan standard of care during pregnancy, and was therefore not administered as part of this study. In addition, WHO policy indicates that vitamin D supplementation is not recommended during pregnancy [28]; therefore, we hope to inform future research and policy recommendations.

### 2.6. Assessment of Pregnancy Outcomes

Pregnancy outcomes, including premature birth, stillbirth, normal delivery, molar pregnancy, and infant anthropometric variables were evaluated immediately after delivery. Gestational age at delivery was defined in weeks; preterm delivery defined as less than 37 weeks of gestation. Weight for age *z*-score (WAZ), length for age *z*-score (LAZ), and weight for length *z*-score (WLZ) were determined for infant anthropometry based on WHO standards; underweight was defined at WAZ < −2, stunted at LAZ < −2, and wasted at WLZ < −2 [29].

### 2.7. Statistical Analysis

Univariate analyses were performed for maternal characteristics at enrollment (age, gestational age, Hb levels, and prevalence of malaria and helminth/protozoan infections) and newborn characteristics (gender, birth weight, and anthropometric data), including means and standard deviations for continuous variables. Anthropometric *z*-scores were computed for primary newborn outcomes using WHO International Growth References (version 3.2.2, January 2011), and WHO cut-off standards were used for categorization as underweight, stunted, and wasted. There is limited consensus on optimal 25(OH)D levels for potential extraskeletal benefits; in our study, we defined vitamin D deficiency and vitamin D insufficiency as plasma 25(OH)D below 50 nmol/L and 75 nmol/L, respectively, based on the Endocrine Society guidelines [30]. Vitamin D quintiles were generated based on the distribution in this population, and birth outcomes were compared between them. Binomial regression was conducted to calculate risk ratios and 95% confidence intervals for the independent variable plasma 25(OH)D levels at enrollment with indicators of maternal nutritional status (anemia, malaria, and protozoan/helminth infection) and birth outcomes. Log-Poisson regression was used to model birth outcomes with the dependent variable when the binomial model failed to converge [31,32]. All multivariate models controlled for maternal age at enrollment and gestational age at delivery. In addition, we compared means of normally distributed plasma 25(OH)D concentrations between malaria infected/uninfected and helminth or protozoan infected/uninfected using a *t*-test for continuous data and chi-squared test for stratified vitamin D status. All statistical analyses were two-sided, and differences were considered significant at *p* ≤ 0.05.

We controlled for confounding variables using the method proposed by Rothman and Greenland by evaluating all known or potential risk factors for the outcome as covariates in the multivariate model [33]. Variables that changed the main effect estimate of interest by >10% were retained in the final multivariate model. We examined test assumptions, which demonstrated that there would not be any significant violations. Statistical analyses were performed using SAS software (version 9.4; SAS Institute, Cary, NC, USA).

## 3. Results

### 3.1. Varying Maternal and Infant’s Plasma 25(OH)D Concentrations

Plasma 25(OH)D concentrations were available for 63 HIV-uninfected pregnant women enrolled at baseline. Characteristics of these 63 women are illustrated in Table 1. The following samples were available for analysis at each time point: ANC-1, *n* = 63; ANC-2, *n* = 38; ANC-3, *n* = 39; ANC-4, *n* = 35; delivery maternal venous, *n* = 56; cord blood, *n* = 43. At enrollment, the mean maternal age (± SD) was 22.5 ± 6.6 years, with a gestational age of 19.9 ± 5.7 weeks. At enrollment, 47.5% of pregnant women (*n* = 28) had anemia (Hb < 11.0 g/dL). Thirty-nine percent (*n* = 24) of pregnant women tested positive for malaria infection by qPCR at enrollment, while 45% (*n* = 19) tested positive for either a pathogenic protozoan or helminth infection (protozoans and helminths tested are specified in Table 1).

**Figure 1:**
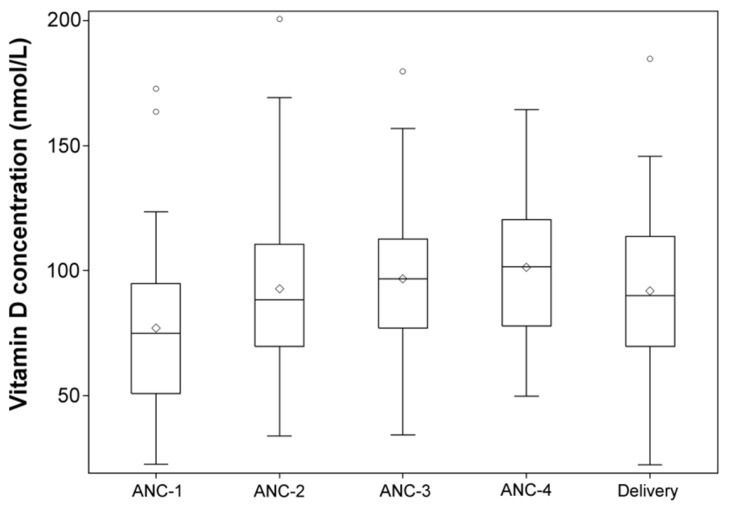
Maternal plasma 25(OH)D concentration over the gestation period visits and delivery (*p* > 0.05).

At their first ANC visit (ANC1), 20.6% (*n* = 13) of mothers had deficient (<50 nmol/L) plasma 25(OH)D concentrations, while 50.8%, (*n* = 32) had insufficient (<75 nmol/L) plasma 25(OH)D concentrations. As shown in Figure 1, no statistically significant differences were found when comparing mean plasma 25(OH)D concentrations across the different ANC visits. Maternal plasma 25(OH)D concentrations at enrollment were evaluated by season of enrollment, and differences were not found to be statistically significant. Plasma 25(OH)D concentrations in cord blood were also measured, and we found 30% (*n* = 13) with deficient levels (<50 nmol/L), and 74.4% (*n* = 32) with insufficient concentration (<75 nmol/L). Mean maternal plasma 25(OH)D levels at delivery were highly correlated between mothers in venous and their corresponding infant’s cord blood levels (*R*^2^ = 0.52, *p* < 0.0001).

### 3.2. Newborns’ Anthropometric Measurements

Characteristics of the newborns to the mothers in the study are shown in Table 1. Out of 54 newborns with available data, 42.6% (*n* = 23) were female and had an average birth weight (±SD) of 3.3 ± 0.4 kg. Mean length and head circumference (±SD) were 49.0 ± 2.7 cm and 35.4 ± 1.5 cm, respectively. However, we observed that there were 18.4% (*n* = 9) infants born at <37 weeks at an average (±SD) gestational age of 39.2 (±2.8) weeks. WHO *z*-scores were calculated based on the infant’s anthropometric measurements at delivery; 15.1% (*n* = 8) were stunted, 12.0% (*n* = 6) were wasted, and 5.7% (*n* = 3) had a BMI *z*-score of < −2.

### 3.3. Protozoa and Helminth Infection Status of Mothers Is Not Associated with Plasma 25(OH)D Levels in Mothers or Their Infants

**Figure 2:**
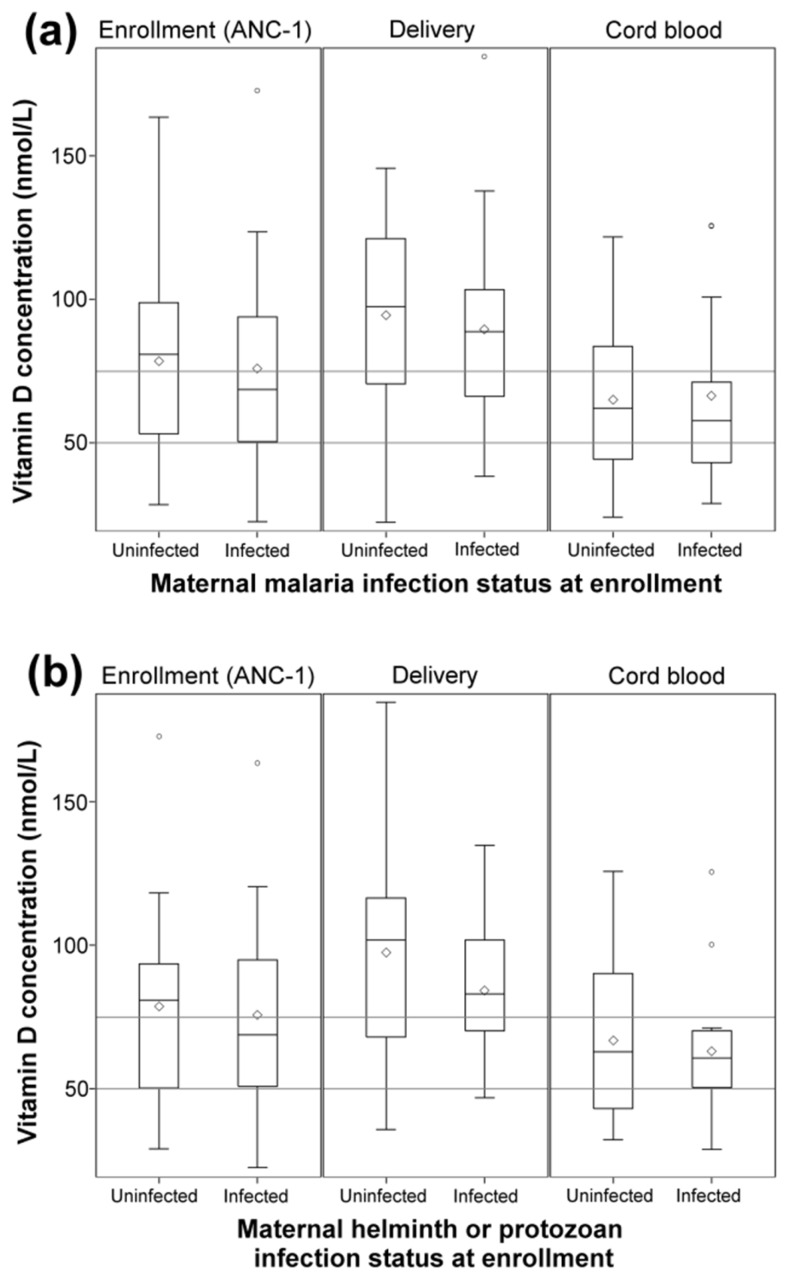
Maternal plasma 25(OH)D concentrations at enrollment (ANC-1), delivery (in venous), and cord blood by (**a**) maternal peripheral malaria infection at enrollment status; and by (**b**) maternal helminth or protozoan infection status at enrollment. Maternal malaria infection status was based on qPCR diagnosis at enrollment. Means were compared using *t*-test with significance set at *p* ≤ 0.05. Horizontal lines show plasma 25(OH)D levels for insufficiency (<75 nmol/L) and deficiency (<50 nmol/L).

As a large number of mothers in this study had malaria (39%), helminth or other protozoan infections (45%) during pregnancy, we determined whether there was any correlation between plasma 25(OH)D concentrations and infection status in mothers at enrollment, delivery, or in infant cord-blood. As illustrated in Figure 2a, there were no significant differences in plasma 25(OH)D concentrations relative to malaria infection status. Interactions of plasma 25(OH)D concentrations with helminth/protozoan infection status were evaluated, and no significant interaction was found (Figure 2b).

### 3.4. Deficient Maternal Plasma 25(OH)D Levels Are Associated with Adverse Pregnancy Outcomes

We next performed a multivariate analysis to determine if maternal plasma 25(OH)D levels at enrollment were associated with adverse pregnancy outcomes. No association was observed between plasma 25(OH)D levels and preterm birth. Both weight-for-age and weight-for-length *z*-scores increased by 0.01 and 0.02 units, respectively, with each nmol/L higher concentration of plasma 25(OH)D (*p* < 0.05) as shown in Table 2. Within this population subset, newborns were over four times as likely to be stunted at birth when born to a mother with a deficient plasma 25(OH)D level at enrollment (RR, 4.4 (CI, 1.0–18.6), *p* = 0.04) (Table 3). Newborns were also more likely to be born at <37 weeks of gestation (preterm birth) when born to mothers with deficient plasma 25(OH)D at enrollment (RR, 5.4 (CI, 1.1, 25.3), *p* = 0.03).

## 4. Discussion

This study sought to determine the association between maternal plasma 25(OH)D concentrations and pregnancy outcomes in rural western Kenya—a region where malaria transmission is holoendemic. We evaluated the concentrations of maternal plasma 25(OH)D during gestation through to delivery, and determined the association of maternal plasma 25(OH)D levels with pregnancy outcomes and infant anthropometric measurements at birth. Our study findings report that both maternal plasma 25(OH)D deficiency and insufficiency levels are evident in the study population. Furthermore, maternal plasma 25(OH)D deficiency levels at enrollment were associated with premature delivery and stunting among mothers and their children, respectively, from malaria holoendemic areas. These data analyses are exploratory and hypothesis-generating in nature due to limited data availability in Equatorial Africa on maternal plasma 25(OH)D levels and birth outcomes.

Multiple measurements and determination of plasma 25(OH)D concentrations throughout pregnancy in a rural area endemic for malaria in Kenya revealed that at enrollment, 51% of the pregnant women in the cohort had plasma 25(OH)D insufficiency, and 21% were deficient. This prevalence is similar to earlier results from sub-Saharan Africa, with similar abundance of sunlight throughout the year. For example, a study in Tanzania found that nearly 40% of pregnant women had vitamin D insufficiency (defined as <80 nmol/L) [19]. In contrast, a previous study from the Western Hemisphere reported higher 25(OH)D concentrations as pregnancy progresses [34]. 

The majority of infants (70%, *n* = 38) born to the pregnant women enrolled in this cohort were plasma 25(OH)D insufficient. This finding is in line with previous study findings reporting high prevalence of vitamin D insufficiency and deficiency in cord blood and which found that differences in ethnicity, skin color, and lack of maternal supplementation contributed to vitamin D insufficiency [35]. Similar to a study by Jacquemyn et al. [9], we found a strong positive correlation between maternal plasma 25(OH)D concentrations at delivery and umbilical cord blood plasma 25(OH)D concentrations. This suggests that supplementation with vitamin D during pregnancy could improve 25(OH)D concentrations of their neonates. A recent study by Wagner et al. [36] reported on a randomized control trial to test Vitamin D_3_ supplementation during pregnancy in a US cohort. They found that increased serum concentrations of 25(OH)D decreased the risk of pre-term birth. 

Several studies have shown lower levels of vitamin D status among individuals infected with a number of different pathogens [21,22,23] compared to those without infection. However, we observed that plasma 25(OH)D levels in pregnant women with and without malaria and other helminth infections in this study were not significantly different. More studies with a larger sample size are needed to clarify the potential role of parasitic infections in vitamin D metabolism in women and children.

This study reports that infants born to women with plasma 25(OH)D deficiency had a four-time higher risk of being stunted at birth using the WHO standards. Several previous studies have examined the association between 25(OH)D concentration, vitamin D intake, and supplementation and anthropometric measurements at birth, with contrasting results [3]. A study by Marya et al. [37] reported findings depicting the benefit of vitamin D supplementation on birth length in India. Therefore, given our findings, there may be a need to carry out cohort studies with adequate sample size and detailed longitudinal assessments of vitamin D status throughout pregnancy, and clinical trial studies of vitamin D supplementation in populations at risk of maternal vitamin D deficiency and the associated adverse effects.

Observed variations in 25(OH)D concentrations among different mothers and their children may be attributed to variation in skin pigmentation, diet, and clothing habits [20]. This observation could be supported by earlier findings showing varying 25(OH)D concentrations in mothers and their children [35,38,39,40]. Our study did not collect data on risk factors influencing 25(OH)D concentrations in the population, including clothing habits, outdoor activities, and dietary habits. It is also possible that individual physiological demand for vitamin D may impact its bioavailability [41,42].

Although infants typically acquire 1,25-dihydroxyvitamin D (the bioactive vitamin D metabolite) from their mothers during gestation through trans-placental transfer [11,43,44], this may vary depending on maternal age and health [5,41]. For example, elderly mothers aged >40 years tend to have high plasma 25(OH)D concentrations, but give birth to children with reduced 25(OH)D concentrations [18,45], probably due to their bodies’ high physiological demand. Additional 1,25-dihydroxyvitamin D is produced from the placenta and the decidua, though the extent of it has not been completely elucidated [46].

In earlier work, findings have shown that maternal plasma 25(OH)D concentration may be a critical determinant of child growth, morbidity, and mortality in sub-Saharan Africa [3,40,41,42]. Data from this study adds to this growing literature demonstrating the potential importance of vitamin D in neonatal growth. However, limitations of this study, including small sample size and lack of additional data on outdoor activity, calcium intake, and measurement of only vitamin D should be noted. In addition, a study by Ettyang et al. reported a high prevalence of vitamin A deficiency in lactating mothers in Western Kenya [47], which could also be another factor driving adverse health outcomes observed in our cohort. Vitamin A supplementation does not occur routinely during pregnancy as part of the prenatal care given in Kenya, so we cannot rule out potential interaction between vitamin A deficiency and vitamin D deficiency. 

Recent clinical trial results suggest that the use of vitamin D supplementation during pregnancy can improve health outcomes in newborns [48,49]; however, results have been mixed, and recommended doses for optimal health vary [2]. A larger, well-designed randomized controlled trial of vitamin D supplementation with an appropriate physiological dose and duration starting early in pregnancy is needed to determine how to best address vitamin D deficiency in rural Kenya, and to assess whether vitamin D supplementation is a missing piece of our current child health care package of interventions.

## Figures and Tables

**Table 1:** Characteristics of the study population, enrollment through to birth.

Variable	Mean ± SD or % (*n*)
**Maternal characteristics at enrollment ANC-1 (*N* = 63)**	
Maternal age, years	22.5 ± 6.6
Gestational age, weeks	19.9 ± 5.7
Body mass index, kg/m	22.9 ± 2.9
Anemia (Hb < 11.0 g/dL)	47.5% (28)
Severe anemia (Hb < 8.5 g/dL)	6.8% (4)
Maternal malaria infection (by qPCR)	38.7% (24)
Maternal pathogenic protozoan ^1^ or helminth infection ^2^	45.2% (19)
Plasma 25(OH)D, nmol/L	77.0 ± 31.5
Plasma 25(OH)D, deficient (<50 nmol/L)	20.6% (13)
Plasma 25(OH)D, insufficient (<75 nmol/L)	50.8% (32)
**Maternal characteristics throughout gestation**	
ANC-2 Visit (*N* = 38)	
Gestational age, weeks	23.6 ± 5.7
Plasma 25(OH)D, nmol/L	92.7 ± 32.3
ANC-3 Visit (*N* = 39)	
Gestational age, weeks	27.2 ± 6.2
Plasma 25(OH)D, nmol/L	96.6 ± 29.5
ANC-4 Visit (*N* = 35)	
Gestational age, weeks	30.4 ± 5.7
Plasma 25(OH)D, nmol/L	101.3 ± 28.1
Plasma 25(OH)D in venous blood at delivery, nmol/L	91.9 ± 32.6
**Newborn characteristics (*N* = 54)**	
Female	42.6% (23)
Gestational age at birth, weeks	39.2 ± 2.8
Preterm (<37 weeks of gestation)	18.4% (9)
Birth weight, kg	3.3 ± 0.4
Length, cm	49.0 ± 2.7
Head circumference, cm	35.4 ± 1.5
Plasma 25(OH)D in cord blood, nmol/L	64.9 ± 26.4
Plasma 25(OH)D, deficient (<50 nmol/L)	30.2% (13)
Plasma 25(OH)D, insufficient (<75 nmol/L)	74.4% (32)

^1^ Protozoans tested: *Entamoeba histolytica* and *Giardia lamblia*; ^2^ Helminth infections tested: hookworm, *Trichuris trichiura*, *Ascaris lumbricoides*, *Schistosoma mansoni*. 25(OH)D: 25-hydroxyvitamin D; ANC: antenatal clinic; Hb: hemoglobin; qPCR: quantitative polymerase chain reaction.

**Table 2:** Maternal Plasma 25(OH)D Concentrations ^1^ and Pregnancy Outcomes.

Outcome	Mean Difference (SE) ^2^	*p*
Gestational age at birth, weeks	0.01 (0.01)	0.41
Birth weight, grams	3.0 (1.6)	0.06
Weight-for-age *z*-score (WAZ)	0.01 (0.00)	0.035 *
Length-for-age *z*-score (LAZ)	0.00 (0.01)	0.70
Weight-for-length *z*-score (WLZ)	0.02 (0.01)	0.02 *
BMI *z*-score	0.01 (0.01)	0.14

Data are no. of offspring with the outcome/no. of offspring assessed (% of offspring with the outcome). SE, standard error. ^1^ Maternal plasma 25(OH)D measured at enrollment; ^2^ Data are the mean outcome difference per 1 nmol/L increase of plasma 25(OH)D concentration. The mean differences and corresponding *p* values were estimated from linear regression models; * Significant difference at *p* ≤ 0.05 level.

**Table 3:** Low Maternal Vitamin D at Enrollment and Adverse Pregnancy Outcomes.

	Maternal Plasma 25(OH)D Level ^1^		Bivariate Analysis		Multivariate Analysis ^2^	
	Low (<50 nmol/L)	Adequate (≥50 nmol/L)				
Outcome	*n/N* (%)	*n/N* (%)	RR (95% CI)	*p* ^3^	RR (95% CI)	*p* ^3^
Preterm birth (<37 weeks)	4/10 (40.0)	5/39 (12.8)	3.12 (1.02, 9.53)	0.06	5.36 (1.13, 25.33)	0.03 *
Stunted (LAZ < −2)	4/10 (40.0)	4/43 (9.3)	4.30 (1.29, 14.32)	0.03 *	4.39 (1.04, 18.55)	0.04 *
Wasted (WLZ < −2)	1/8 (12.5)	5/42 (11.9)	1.05 (0.14, 7.83)	0.96	1.12 (0.12, 10.36)	0.92
BMI *z*-score (<−2)	1/10 (10.0)	2/43 (4.7)	2.15 (0.22, 21.44)	0.54	1.76 (0.15, 19.97)	0.65

Data are no. of offspring with the outcome/no. of offspring assessed (% of offspring with the outcome). CI, confidence interval; RR, risk ratio; ^1^ Maternal vitamin D measured at enrollment; ^2^ Multivariate model for preterm birth outcome was adjusted for maternal age, body mass index (BMI), and gestational age (all at enrollment); all other multivariate models were adjusted for maternal age and BMI at enrollment, and gestational age at delivery; ^3^ *p* values are derived from log-binomial regression models; * Significant difference at *p* ≤ 0.05 level.
